# Alzheimer’s disease and infections, where we stand and where we go

**DOI:** 10.1186/s12979-014-0026-4

**Published:** 2014-12-17

**Authors:** Roberto Monastero, Calogero Caruso, Sonya Vasto

**Affiliations:** Department of Experimental Biomedicine and Clinical Neurosciences, University of Palermo, Palermo, Italy; Department of Pathobiology and Medical and Forensic Biotechnologies, University of Palermo, Palermo, Italy; National Center for Research, Institute of Biomedicine and Molecular Immunology, Palermo, Italy; Department of Science and Biological, Chemical and Pharmaceutical Technologies, Institute of Biomedicine and Molecular Immunology, Palermo, Italy

**Keywords:** Alzheimer’s disease, Herpes viruses, Infections, Inflammation

## Editorial

Alzheimer’s disease (AD) is a progressive neurological disorder, which represents the most common form of dementia, one of the major causes of disability in later life. Age is the greatest risk factor for AD, which typically affects people aged 65 years and over, with an age-standardised prevalence of 4.4 [1]. However, AD is not a normal part of ageing and advanced age alone does not justify the disease. Several pathways have been implicated in AD pathophysiology, the most described is the neurodegenerative one, which lead to the brain accumulation of beta-amyloid and neurofibrillary tangles, aggregations of hyperphosphorylated tau protein, macroscopically resulting in brain atrophy due to neuronal death [2]. These pathological hallmarks of AD have been recently incorporated in the new recommendations on diagnostic guidelines for AD, which describe different stages of the disease, including its preclinical and symptomatic pre-dementia phases [3].

Genetics accounts for less than 3% of AD, familiar AD at early onset, resulting from mutations in three genes, i.e. APP, PS1 and PS2. Furthermore, the Apolipoprotein E4 (ApoE4) genotype is the only, robust, susceptibility gene for AD [2], although meta-analysis and genome scanning have revealed several susceptibility loci with low odds ratios [4,5]. Overall, multiple gene-gene and environment interactions cause AD; however, various risk factors differently act throughout ageing [2,6]. Large data have been collected in the last two decades regarding the putative role of vascular disease, including systemic atherosclerosis, high blood pressure, diabetes, high level of cholesterol, tobacco smoking, as well as other vascular risk factors, as pathogenetic cause of AD [6-8]. However, a central role for systemic inflammation has been claimed also taking into account previously reported data, traumatic brain injury and oxidative stress [9-13]. Indeed, only a small percentage of people aged 80 years or over has a pure neurodegenerative AD, and mixed dementia with vascular and/or inflammatory components are present [14]. Peripheral inflammation is indeed present in early stage of AD and is higher than that observed during non-pathological ageing [13]. Moreover, an altered inflammatory regulation is also present in Mild Cognitive Impairment (MCI), the intermediate stage between the expected cognitive decline of normal ageing and the more serious decline of dementia [15], and correlates with the progression to AD [13].

Accordingly, acute episodes of systemic inflammation with increased levels of inflammatory mediator tumor necrosis factor-alpha, which are associated with AD [5], have been shown to be associated with long-term cognitive decline in a prospective cohort study of subjects with AD [16]. The missing link between central neuro-inflammation and peripheral inflammatory state might be represented by infectious factors [17].

The possibility of an infectious aetiology for AD has been repeatedly proposed over the past three decades, suggesting the role of viral and bacterial chronic infections as causative inflammatory pathway for AD. Concerning bacterial infections, data from a recent meta-analysis demonstrated that Spirochetal or Chlamydophila Pneumoniae infections were strongly associated with AD [18].

More interestingly, the role of chronic inflammation in periodontal disease (PD) has been suggested over the last decade as a potential risk factor for AD [17,19-21]. In particular, researchers from US found that antibody levels to specific oral pathogens were significantly increased at baseline serum draw in subjects who lately developed MCI or AD, thus suggesting that PD could potentially contribute to the risk of AD [21]. In cases of severe PD, pro-inflammatory molecules may induce a systemic inflammation and may, therefore, access the brain via systemic circulation. Pro-inflammatory molecules, derived locally from periodontal tissue, may stimulate trigeminal nerve fibres, leading to an increase in the number of brain cytokines. These cytokines may act on the already primed glial cells, resulting in an amplified reaction and possible progression of AD [18,20].

The concept of a viral role in AD, specifically of herpes simplex virus type 1 (HSV-1), was first proposed several decades ago. However, it was only in 1991 that by polymerase chain reaction it was looked for HSV-1 DNA in autopsy brain specimens. In all specimens from 8 AD patients and 6 normal individuals (from temporal, frontal and hippocampal lobes), the authors found viral sequences (22). It was postulated that factors such as number or expression of viral genes and host susceptibility might be related to incidence of AD [22,23].

HSV-1 is a ubiquitous virus that affects more than 80% of people over 65 worldwide. It is a neurotropic double-stranded DNA virus that primarily infects epithelial cells of oral and nasal mucosa, where virus undergoes lytic replication; the newly produced viral particles may enter sensory neurons and, by axonal transport, reach the trigeminal ganglion where they usually establish a latent infection. The trigeminal ganglion neurons also project to the trigeminal nuclei located in the brainstem. From here, neurons project to the thalamus to finally reach the sensory cortex. This is the path through which the reactivated virus may reach the central nervous system (CNS), where it may cause acute neurological disorders like encephalitis or a mild, clinically asymptomatic, infection, or establish lifelong latent infection. It has been proposed that virus is normally latent in many elderly brains but reactivates periodically, as in the peripheral nervous system, under certain conditions, for example stress, immunosuppression, and peripheral infection, causing cumulative damage and eventually development of AD [17,23,24]. Thus, elderly immunosenescence might be responsible for its reactivation [17]. Several epidemiological studies have shown, using serological data, an association between systemic infections and cognitive decline, with HSV1 particularly implicated [17,24,25]. A very recent Swedish nested case–control study showed that the presence of anti-HSV-1 IgG antibodies doubled the risk for AD in persons for whom plasma was collected more than 6.6 years before the AD diagnosis [26]. Of interest, this risk increased in subject aged over 60 years and among females. Another study from Italy reported that elevated serum HSV-1 antibody titres correlated with cortical grey matter volume [27].

It is interesting to note that other herpes viruses share the ability to become latent in the infected host and eventually latently infect neurons. However, investigations focused on different viruses of the herpes family, such as human cytomegalovirus (CMV), Epstein-Barr virus (EBV) or human herpes virus 6 (HHV-6) in AD are limited [17]. A recent work showed that increased CMV antibody levels were present in the elderly who developed clinical AD during a five years follow-up [28]. In a study of deceased and autopsied subjects from a clinical-pathological community cohort, the authors found associations of CMV-related immunologic and virologic characteristics with AD neuropathology and additional trends toward associations with clinical diagnosis [29]. Nonetheless, these findings could equally well be explained by an indirect effect since reactivation of HSV-1 is associated with CMV and age, perhaps via CMV-induced immunosenescence [30,31]. On the other hand, a few data present in literature concerning the serological association between EBV or HHV-6 and AD could be explained by a similar indirect effect. Both HSV-1 reactivation and EBV and HHV-6 antibody stimulation can, in fact, be triggered by T immunosenescence that is stronger in AD than in control elderly [32]. As an alternative, but not mutually exclusive, possibility, EBV and HHV-6 titles might indicate a systemic inflammation responsible for HSV-1 reactivation [33].

Indeed, and as reviewed by Itzhaki [23], there is evidence for direct possible pathophysiological mechanisms in AD only for HSV-1 since reactivated HSV-1 can cause direct and inflammatory damage in CNS. Implicating HSV-1 further in AD is the discovery that HSV-1 DNA is specifically localized in amyloid plaques in AD. In addition, data by several groups show that HSV-1 infection of cells in culture causes formation of β-amiloid, datum initially found by Wozniak et al., [34] and of AD-like tau, datum initially found by Zambrano et al. [35]. Other relevant, harmful effects of infection include the following: dynamic interactions between HSV-1 and amyloid precursor protein (APP), which would affect both viral and APP transport [23].

As previously stated, findings from a genome-wide association study in a large cohort of patients with AD showed that a limited set of genes were associated with the disease [4]. Licastro and co-workers [17,36] suggest that the polymorphism association in some of these genes is consistent with a non-conventional interpretation of AD aetiology. These data suggest that differential genetic backgrounds in genes regulating immune defences against herpes viruses are associated with age-related cognitive deterioration and AD. Cycles of virus latency/infections may therefore contribute to neurodegeneration associated with AD in genetically predisposed elderly, leading to neuronal loss, inflammation and amyloid deposition.

However, only a few prospective cohort studies have confirmed the role of viral and bacteria infections in AD. Overall, available data suggest a link between chronic infections and increased risk for AD, possibly through a low-grade, chronic infection and inflammation in individuals who have inherent susceptibility traits. However, the majority of researches conducted have been cross-sectional, observational studies, which include relatively small hospital-based samples with inherent problems of selection and residual confounding. Accordingly, further prospective, population-based studies conducted in large cohorts investigating the link between infection and AD are warranted, taking into account APOE typing because of its involvement both in AD and chronic infections [2,37,38]. In any case, successful treatment of chronic infections is a challenging but mandatory goal to improve the quality of life in the elderly.
